# Electrical Impedance Spectroscopy for Monitoring Chemoresistance of Cancer Cells

**DOI:** 10.3390/mi11090832

**Published:** 2020-08-31

**Authors:** Lexi L. Crowell, Juan S. Yakisich, Brian Aufderheide, Tayloria N. G. Adams

**Affiliations:** 1 Department of Chemical and Biomolecular Engineering, University of California-Irvine, Irvine, CA 92697, USA; crowelll@uci.edu; 2 Sue & Bill Gross Stem Cell Research Center, University of California Irvine, Irvine, CA 92697, USA; 3 Department of Pharmaceutical Sciences, Hampton University, Hampton, VA 23668, USA; juan.yakisich@hamptonu.edu; 4 Department of Chemical Engineering, Hampton University, Hampton, VA 23668, USA; brian.aufderheide@hamptonu.edu

**Keywords:** electrical impedance spectroscopy, dielectrophoresis, impedance, capacitance, microfluidics, electrokinetics, cell phenotype, cancer cells

## Abstract

Electrical impedance spectroscopy (EIS) is an electrokinetic method that allows for the characterization of intrinsic dielectric properties of cells. EIS has emerged in the last decade as a promising method for the characterization of cancerous cells, providing information on inductance, capacitance, and impedance of cells. The individual cell behavior can be quantified using its characteristic phase angle, amplitude, and frequency measurements obtained by fitting the input frequency-dependent cellular response to a resistor–capacitor circuit model. These electrical properties will provide important information about unique biomarkers related to the behavior of these cancerous cells, especially monitoring their chemoresistivity and sensitivity to chemotherapeutics. There are currently few methods to assess drug resistant cancer cells, and therefore it is difficult to identify and eliminate drug-resistant cancer cells found in static and metastatic tumors. Establishing techniques for the real-time monitoring of changes in cancer cell phenotypes is, therefore, important for understanding cancer cell dynamics and their plastic properties. EIS can be used to monitor these changes. In this review, we will cover the theory behind EIS, other impedance techniques, and how EIS can be used to monitor cell behavior and phenotype changes within cancerous cells.

## 1. Introduction

Cancer is a life altering disease that affects over 15 million people in the United States, and new cases are expected to rise to 19 million people by 2024 [1]. Breast, lung, and prostate are the most newly diagnosed cases in 2020, reaching a number of roughly 700,000 new cases [1]. Some cancers have a long latent period allowing for early diagnosis [2]. However, once cancer becomes chemoresistant, it is difficult to treat, therefore, contributing to high mortality rates. Chemoresistance accounts for more than 90% of cancer reoccurrence and high mortality rates, due to few methods to assess drug efficacy [3,4]. Current methods such as polymerase chain reaction (PCR), real-time PCR, blotting, flow cytometry, and next generation sequencing are used to identify gene expression related to specific biomarkers to monitor the chemoresistance of cancer cells [5]. Disadvantages to these methods include they require trained technicians, long preparation times (~48 h per assessment), and they lack continuous monitoring. Additionally, these methods typically have limited ability to assess the phenotypic changes associated with drug chemoresistance. Therefore, there is demand for a simple, quick, and continuous method for monitoring the chemoresistance of cancer.

To better study chemoresistance, alternative technologies are needed to (1) enable the detection of drug resistant cancer cells before symptoms reappear, (2) monitor disease progression, and (3) evaluate patient response to treatment. Understanding when, how, and why cancer becomes chemoresistant will further advance our knowledge of cancer biology. Cancer cell dynamics can be assessed based on their heterogeneity, stemness, and plasticity. Cancer cell plasticity refers to the ability of a cell to undergo reversible molecular and phenotypic change in response to their microenvironment [6,7,8]. This is a key factor that limits the effectiveness of chemotherapy drugs [8,9]. Plasticity is a property of all cancers and it contributes to heterogeneity. The exact time scale at which a phenotypic change occurs within the body, is puzzling, making it difficult to treat cancer cells once they become chemoresistant, especially after metastasis occurs [8,10,11]. Currently, two- and three-dimensional (2D, 3D) cell culture models are used to understand the cellular dynamics involved in plasticity; however, these models may not be an accurate predictor of cancer cell chemoresistance [9]. These models are limited because they do not account for experimental information about cancer cells microenvironment, vasculature, or other intrinsic and extrinsic determinants that contribute to plasticity and heterogeneity. Many studies corroborate the need to understand cancer cell dynamics and their interactions with drugs, toxins, and the microenvironment [11]. Ideally, a technique for continuous monitoring of cancer cells and chemoresistance would be beneficial, allowing for short assessment time and low cost. One option is to utilize electrical impedance spectroscopy to measure the dielectric properties of cancer cells in order to monitor chemoresistance.

**Figure 1:**
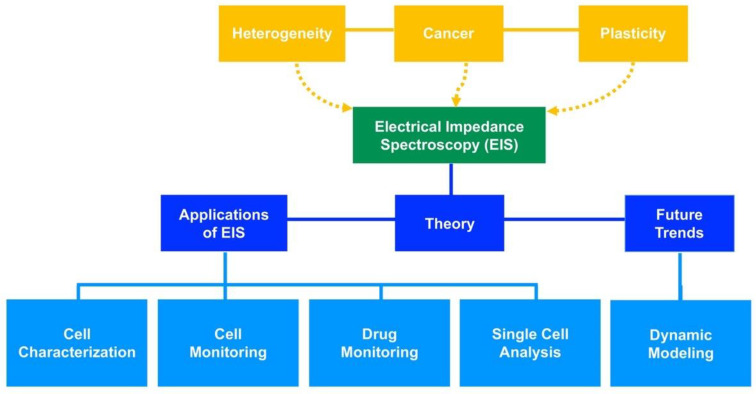
Overview of the main topics covered in this review.

Electrical impedance spectroscopy (EIS) is an electrokinetic technique that can be used as a non-invasive, label free approach for the recognition of cancer cells, their dynamics, and chemoresistance [12]. EIS relies on cell polarization generated by an electric field and the interaction of ions along the cell surface [13]. Impedance-based biosensors have been used to distinguish many types of human and mouse cancer cells based on their dielectric properties (impedance and capacitance) [14,15,16,17]. EIS is advantageous because it enables real time measurements of cell dynamics. In this paper, we will review cancer cell plasticity and recent developments of EIS to assess its capability to monitor changes at the molecular level of cancer cells. Several EIS studies completed on cancerous and noncancerous cells are assessed. We will also highlight the benefits of using pharmacodynamic mathematical modeling for cancer cell plasticity. Our goal is to provide insight to the feasibility of detecting plasticity and ultimately monitoring cancer chemoresistance using EIS. Figure 1 outlines the main topics covered in this review.

## 2. Heterogeneity of Cancer Cells

Cancer cells’ heterogeneity is characterized by distinct morphologies, stemness, metabolic activity, genetic, epigenetics and plasticity [18,19,20,21], all factors that contribute to chemoresistance. Plasticity is an interesting dynamic property of all cells defined as a cell’s ability to undergo reversible molecular and phenotypic changes in response to their microenvironment [19]. Visually, plasticity is the fluid transition of one or more cells from one state to another and is a key factor that limits the effectiveness of chemotherapy treatments. It is estimated that plasticity changes occur around day three of in vitro aging [9]. However, within the body it is more difficult to identify when this change occurs.

**Figure 2:**
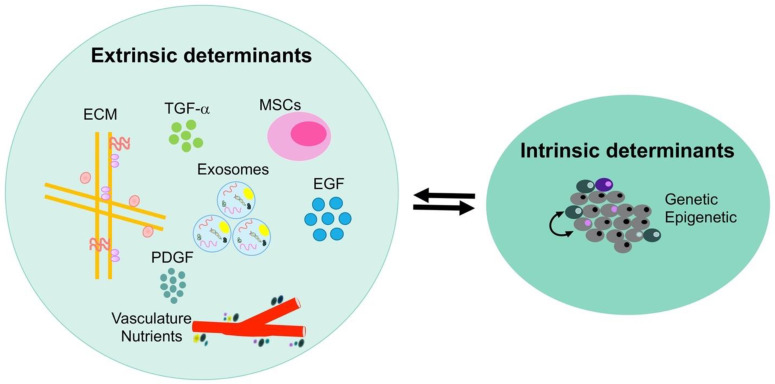
Intrinsic and extrinsic determinants that contribute to cancer cell heterogeneity. The heterogeneity arises from cell intrinsic phenotype (genetics, epigenetics, and cell origin), cell extrinsic determinants (extracellular matrix, vasculature, nutrients, MSCs, exosomes, TGF- α, EGF, and PDGF). Adapted from [4].

Plasticity is driven by intrinsic and extrinsic determinants in the biological activities of cancer cells and the microenvironment illustrated in Figure 2. Intrinsic determinants are impacted by the inherent properties of a cell which contribute to its oncogenic phenotype (i.e., genetic and epigenetic changes) [19,22]. Extrinsic determinants are impacted by the microenvironment surrounding a cell and can be studied by examining secreted exosomes, microvesicles, growth factors (TGF-α, EGF, and PDGF), and stresses such as hypoxia [18,20]. These extrinsic determinants contribute to cancer metastasis, where extracellular vesicles are found in the blood stream of late stage cancer patients. As a result, dissemination of these vesicles accounts for 90% of all cancer related deaths [4]. It should be reiterated that one benefit of EIS is the ability to monitor the dynamics of intrinsic and extrinsic changes that occur in cancer cells.

Different models such as the classical cancer stem cell theory (CCSCT) proposes the existence of a rare subpopulation of cancer stem cells (CSCs) and partially explains the heterogeneity and the chemoresistance found in tumors. The CCSCT is a unidirectional model in which CSCs have the ability to induce phenotypic changes into more differentiated (or more specialized) non-CSCs but non-CSCs cannot differentiate into CSCs. It was generally accepted that eliminating the CSC subpopulation was the key to prevent tumor growth and relapse. However, experimental data failed to fully reconciliate this concept due to the rigidity of the CCSCT model. As a result, alternative and more plastic (bidirectional) models based on cancer stemness were proposed [18]. The stemness phenotype model (SPM) and cancer stem cell plasticity model can be used to predict and study cancer cell dynamics. SPM suggests that all cancer cells have stem cell properties, mostly depending on the microenvironment, while the cancer stem cell plasticity model proposes that cancer cells have the capability to transform between non-CSC and CSC states due to intrinsic and extrinsic determinants [18].

The plastic properties of cancer cells driven by the intrinsic and extrinsic determinants have enormous clinical implications. Two important predictions of SPM were validated experimentally: 1) the ability of cells to undergo interconversion between a non-CSC phenotype and a CSC phenotype was demonstrated in several cancer cell lines, such as breast [23,24,25], colon [24,25,26], lung [27], and glioblastoma [28], and 2) the ability of a single cancer cell to repopulate the heterogeneity of a tumor was recently demonstrated in an in vivo animal model of glioma [29]. It is clear that there is a shift in our understanding of cancer cell biology from the rigid unidirectional model originally proposed by CCSCT to a more plastic bidirectional model. From the clinical perspective, plasticity-based models predict that to cure cancer all cancer cells should be eliminated at once because any single surviving cell has the ability to repopulate the original tumor and to recapitulate the intratumoral heterogeneity and chemoresistance. In support of this hypothesis, a mathematical model predicted that chemotherapeutic elimination of in vitro cultures of heterogeneous cancer cells with interconversion will be effective only if it targets all cancer cell types [30].

This paradigm shift is also important at the experimental level since it changes the way in which cancer cells should be studied to understand the origin of intratumoral heterogeneity and chemoresistance [31]. For instance, establishing techniques for real-time monitoring of plasticity can improve our understanding of cancer cell dynamics and allow for the development of prediction analysis tools to better treat patients.

## 3. Electrical Impedance Spectroscopy (EIS)

Impedance can be used to detect electrical properties of biologics based on the capacitance and electron transfer resistance of a measured sample. In the early 1920′s, two scientists, Frick and Morse, assessed the difference of one dielectric property, membrane capacitance, between malignant tumors and normal breast tissues [32]. It should be noted that other researchers have used impedance sensing to examine healthy and cancerous tissue for characterization [33,34,35] and imaging [36]. However, those studies are beyond the scope of this review. Our focus is on the dielectric properties of cancer cells in suspension, adhered to substrates, or cancer spheroids. These systems are better suited to examine the plasticity and chemoresistance of cancer cells. 

### 3.1. Theory

In the last decade, electrical impedance sensing has emerged as a promising field for cancer detection. Impedance sensing uses a frequency-dependent signal to measure the impedance, inductance, capacitance, and resistance of cells. When an alternating current is applied, the complex impedance, $\tilde{Z}\left(\omega \right)$, of the circuit can be calculated as [13]:

(1) $$\tilde{Z}\left(\omega \right)=\frac{\tilde{U}\left(\omega \right)}{\tilde{I}\left(\omega \right)}={\tilde{Z}}_{RE}+j{\tilde{Z}}_{IM}$$

where $\tilde{U}\left(\omega \right)$ is the voltage, $\tilde{I}\left(\omega \right)$ is the current, ${\tilde{Z}}_{RE}$ is the real part of the complex impedance, ${\tilde{Z}}_{IM}$ is the imaginary part of the complex impedance, $j=\sqrt{-1}$, and *ω* is the angular frequency (ω=2πf). Another measure of impedance is the combination of the magnitude, $\left|\tilde{Z}\right|,$ and the phase shift, θ, given as [13]:

(2) $$\left|\tilde{Z}\right|=\sqrt{{\left({\tilde{Z}}_{RE}\right)}^{2}+{\left({\tilde{Z}}_{IM}\right)}^{2}}$$

(3) $$\theta =\arctan\left(\frac{{\tilde{Z}}_{IM}}{{\tilde{Z}}_{RE}}\right)$$

These fundamental equations can be applied to study the impedance and resistive properties of cancerous cells. Impedimetric techniques can be divided into three categories, namely electric cell–substrate impedance sensing (ECIS), impedance flow cytometry (IFC), and EIS, and each are useful for studying cancer cells.

**Figure 3:**
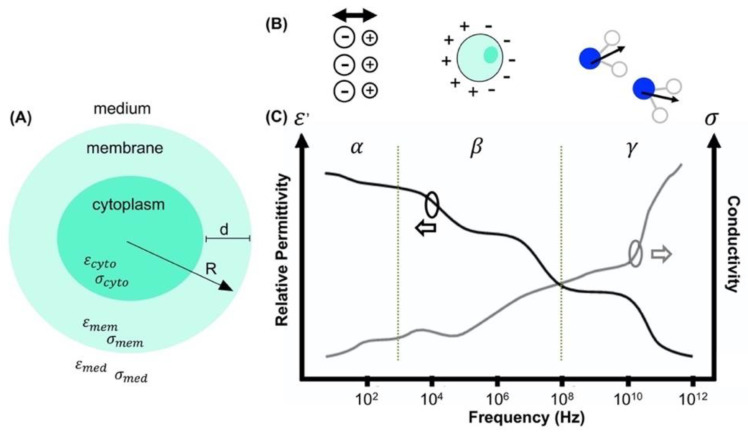
(**A**) Schematic of single shell spherical model for cells [37], (**B**) ionic, interfacial, and dipolar polarization mechanisms [38] associated with (**C**) α, β, and γ dielectric dispersions [38,39], respectively.

Impedance-based techniques use resistor-capacitor theory to model polarization of single cells suspended in low conductivity medium. Cells with a bilayer can be modeled as a single shell sphere containing a conductive sphere with an insulating thin shell which represents the cytoplasm and the plasma membrane, respectively, in Figure 3A [13,37]. This model is described by Maxwell’s mixture theory where the complex permittivity of a cell mixture, $\varepsilon_{mix}$, is given by [13,37]:

(4) $${\tilde{\varepsilon }}_{mix}={\tilde{\varepsilon }}_{med}\frac{1+2\varphi f_{CM}}{1-\varphi f_{CM}}$$

(5) $$f_{CM}=\frac{{\tilde{\varepsilon }}_{cell}-{\tilde{\varepsilon }}_{med}}{{\tilde{\varepsilon }}_{cell}+2{\tilde{\varepsilon }}_{med}}$$

(6) $${\tilde{\varepsilon }}_{cell}={\tilde{\varepsilon }}_{mem}\frac{{\left(\frac{R}{R-d}\right)}^{3}+2\left(\frac{{\tilde{\varepsilon }}_{cyto}-{\tilde{\varepsilon }}_{mem}}{{\tilde{\varepsilon }}_{cyto}+2{\tilde{\varepsilon }}_{mem}}\right)}{{\left(\frac{R}{R-d}\right)}^{3}-\left(\frac{{\tilde{\varepsilon }}_{cyto}-{\tilde{\varepsilon }}_{mem}}{{\tilde{\varepsilon }}_{cyto}+2{\tilde{\varepsilon }}_{mem}}\right)}$$

where ${\tilde{\varepsilon }}_{med}$ is the complex permittivity of the conductive medium, φ is the volume fraction (ratio of cell volume to detection volume), $f_{CM}$ is the Clausius–Mossotti factor, and ${\tilde{\varepsilon }}_{cell}$ is the effective complex permittivity of the cell. Equation (6) accounts for the intrinsic dielectric properties of cells where *R* is the radius, *d* is the thickness of the cell membrane, ${\tilde{\varepsilon }}_{cyto}$ is the complex permittivity of the cytoplasm, and ${\tilde{\varepsilon }}_{mem}$ is the complex permittivity of the membrane. The complex permittivity of the cytoplasm and membrane are given by ${\tilde{\varepsilon }}_{cyto}=\varepsilon_{cyto}+\frac{\sigma_{cyto}}{\omega j}$ and ${\tilde{\varepsilon }}_{mem}=\varepsilon_{mem}+\frac{\sigma_{mem}}{\omega j}$, respectively, where $\varepsilon_{cyto}$ is the permittivity of the cytoplasm, $\sigma_{cyto}$ is the conductivity of the cytoplasm, $\varepsilon_{mem}$ is the permittivity of the membrane, and $\sigma_{mem}$ is the conductivity of the membrane [13,37]. Permittivity is inversely proportional to the complex impedance and describes a cell’s ability to resist the electric field. It decreases as the frequency increases, whereas conductivity increases.

Polarized cells undergo unique polarization mechanisms, as shown in Figure 3B, at distinct dielectric dispersions, which can be separated into three dispersion regions (α, β, and γ) illustrated by Figure 3C. The α-dispersion region is defined below 1 kHz and represents the polarization of ions in the conductive medium [40]. The β-dispersion region is defined from 1 kHz to 100 MHz and polarization is dominated by the cell membrane (lower frequencies) and the cytoplasm (higher frequencies). The γ-dispersion region, which is of least interest when examining cells, is defined from 100 MHz to 100 GHz and supplies information about polarization of water molecules [38,39]. For impedance measurements cells are suspended in conductive medium containing mostly water, sugar, and salt. The dielectric dispersions coupled with model equations are used to obtain cell’s dielectric properties. Impedance measurements can aid in the characterization and monitoring of cancerous cells. The β-dispersion region may reveal characteristics of cancer cell dynamics such as the intrinsic and extrinsic properties, which contribute to cancer cell heterogeneity and phenotype change, therefore indicating chemoresistance. 

**Figure 4:**
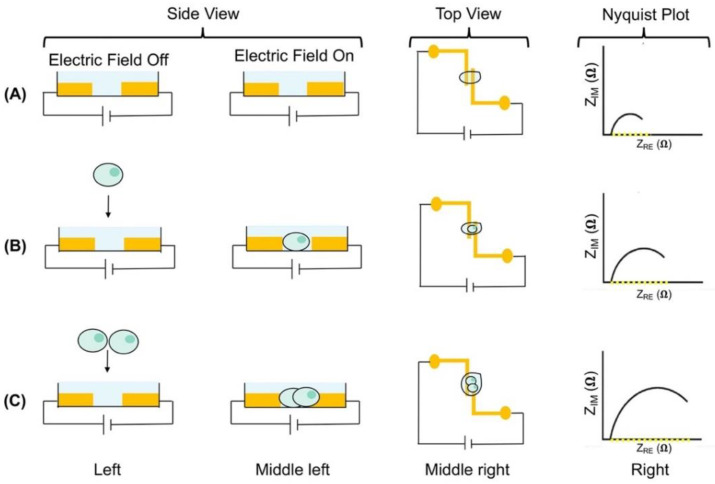
Schematic representation of cell trapping due to electric field polarization for impedance measurements. (**A**) A microfluidic device and conductive medium with the electric field off (left) and the electric field on (middle left). Top view of parallel electrodes with no cells (middle right) yielding lower impedance indicated in Nyquist plot (right). (**B**) A cell placed in the device with the electric field off results in no polarization (left) and with the electric field on polarization occurs and the cell traps between the electrodes (middle left). Top view of parallel electrodes with a cell (middle right) yielding higher impedance indicated in Nyquist plot (right). (**C**) Two cells placed in the device with the electric field off results in no polarization (left) and with the electric field on cells polarize and trap between the electrodes (middle left). Top view of parallel electrodes with the two cells (middle right). Impedance increased due to the presence of two cells indicated in Nyquist plot (right). The impedance with the electric field off is shown on Nyquist plots with yellow dotted line. Electrodes colored dark yellow. Light green circles represent cells and the dark green circles are the nuclei.

To collect impedance data, when the electric field is applied, it will interact with ions available in the conductive medium causing the ions to align around the cell caused by interfacial polarization. The interfacial polarization induces cell movement and is affected by the content and properties of the cell surface [13]. Figure 4 crudely cartoons cell trapping due to electric field polarization and the resulting impedance. Initially, the electric field is off and only the conductive medium is inside the microfluidic device (Figure 4A, left). The electric field is turned on and the impedance is measured to establish a baseline impedance of the conductive medium (Figure 4A, middle left). A top view of the electrodes is included (Figure 4A, middle right) and a lower impedance is measured indicated with the Nyquist plot (Figure 4A, right). When one cell is placed in the microfluidic device with the electric field off no cell polarization occurs (Figure 4B, left). Once the electric field is turned on the cell polarizes and traps between the electrode (Figure 4B, middle left). A top view of the electrodes with the cell trapped is included (Figure 4B, middle right) and a higher impedance is detected due to the cell as indicated in the Nyquist plot (Figure 4B, right). Impedance increases further when more than one cell is trapped between the electrodes, which is indicated in the Nyquist plot (Figure 4C). It is known that applying an electric field at high voltages can permeabilize the cell membrane [41]. To mitigate this, a low voltage should be applied for cell characterizations.

### 3.2. Impedance Spectroscopy of Adhered Cells

The electric cell-substrate impedance sensing (ECIS) technique was the first reported impedance biosensor primarily used to measure adherent cells. ECIS can detect cell attachment, proliferation, and viability based on differences in impedance values. As cells attach to the substrate surface, the impedance increases, adding resistance to the circuit. The impedance value continues to increase as the monolayer completely forms, and plateaus once the electrodes are saturated [42]. ECIS provides the average dielectric signature for a cell population, which is important for continuous real-time monitoring of cells. Adhered cells going through apoptosis have lower impedance values due to the cell membrane being compromised, and it has been found that ECIS can sense when anti-cancer drugs induce apoptosis in cancer cells [14,43]. Thus, impedance can be used to assess cell death and protein secretion during apoptosis. With ECIS it is also possible to detect and monitor motility of adhered cells which is linked to the metastatic state of cancer cells [44]. While ECIS has proven strengths, some disadvantages include the difficultly in single cell analysis, environmental effects, such as temperature or pH, and finding the optimal plating and coating density of cells, which will alter impedance output readings. 

### 3.3. Impedance Spectroscopy of Cells in Suspension

Both impedance flow cytometry (IFC) and electrical impedance spectroscopy (EIS) can be used as techniques to gather impedance data for bulk cell suspensions, clustered cells, or single cells. Fluid flow is incorporated in IFC setups to measure the impedance of cells flowing pass electrodes. The electrodes are planar and located on the bottom of the microchannel [45] or integrated on the top and bottom of the microchannel to extend the height of the electric field increasing sensitivity [46,47]. Additionally, IFC systems may include a constriction in the microchannel, where the cross-sectional area is less than the cell’s cross-sectional area, to further improve detection capability of single cells [48]. When single cells flow through the microchannel constriction the electric field becomes blocked resulting in increased impedance versus when no cells are present. The measured impedance signal is amplified because the measurement area is smaller in the constriction in comparison to planar electrodes in a non-constricted microchannel [48]. EIS, on the other hand, capitalizes on cells being polarized and trapped between planar electrodes located on the bottom of the microchannel [13]. EIS single cell analysis is achieved by altering the electrode geometry such that only one cell is trapped [49,50,51,52,53] and sensitivity is increased by manipulating the applied frequency or the conductivity of the buffer solution. Due to operational differences, the impedance output measured by a flow system versus trapped cells may be different. The main advantage of IFC is high throughput single cell analysis [54,55]. EIS has the advantage of allowing for high throughput whole cell population analysis. IFC and EIS techniques can be used for the characterization [48] and detection of cancer cells [49,55,56,57].

**Figure 5:**
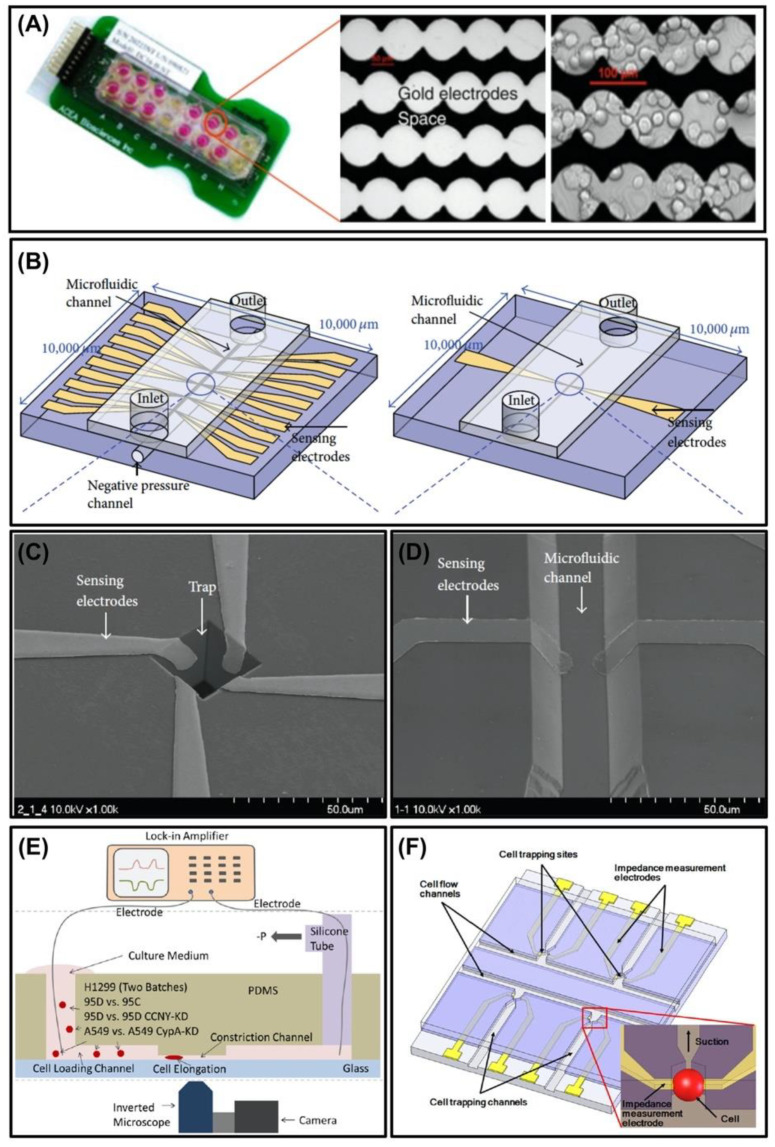
Schematics of (**A**) ECIS microdevice for adhered cells [58] and (**B**) EIS (left) and IFC (right) [59] microdevices for cell suspensions used in impedimetric measurements. Micrographs of planar electrodes for both (**C**) ECIS and (**D**) IFC microdevices [59]. Microdevice schematics of (**E**) IFC system with a microchannel constriction [48] and (**F**) EIS with cell trapping [51] for single cell impedimetric measurements. Reprinted with permission from [48,51,58,59].

Overall, ECIS, EIS, and IFC can be used to assess cell dielectric properties (permittivity, impedance, capacitance), different cell processes (adhesion, proliferation, spreading, motility, and apoptosis), cell concentration and cytological stages. Figure 5 illustrates these impedimetric microfluidic devices. The main advantages to these techniques are their small size (microscale measurements), low cost of device fabrication, and no microscope imaging required (increases portability and decreases experimental time). All these aspects are important in examining cancer cell dynamics.

## 4. Applications of Impedance Spectroscopy for Cancer Cells 

### 4.1. Cell Characterization 

The distinct dielectric properties of cancer cells and their unique cellular events suggests that they can be monitored with label-free techniques. EIS has been used to quantify the impedance, permittivity, capacitance, and conductivity as well as discern differences in a variety of cancer cells. Kang et al. [57] distinguished between human prostate cells (RWPE-1) and human prostate cancer cells (PC-3) using EIS. The microfluidic device employed used pneumatic pressure and a tunnel structure to trap single cells in suspension on the sensing electrodes. The width of the tunnel was designed to be smaller than the cell diameter (~30 µm) to increase the sensitivity of the EIS measurements by containing the electric field within the cell. The β-dispersion region was tested (100 Hz–1 MHz) and a distinct 29.5% difference in the impedance of RWPE-1 and PC-3 was found at 8.7 kHz. Qiao et al. [50] used EIS measurements to differentiate human breast cells (MCF-10A), early stage breast cancer cells (MCF-7), and invasive breast cancer cells (MDA-MB-231). The microfluidic system used needle electrodes to measure membrane resistance, membrane capacitance, and intracellular resistance at 37 °C. The trends for the dielectric properties were variable MDA-MB-231 cells had the highest intracellular resistance (1711.1 Ω) followed by MCF-10A cells (1542.2 Ω) and MCF-7 cells (1474.5 Ω). For membrane capacitance MCF-10A cells had the highest value (1.3 nF) followed by MDA-MB-231 cells (0.7 nF), and MCF-7 cells (0.5 nF). Additionally, Zhao et al. [48] used an IFC single cell microdevice system with a constriction, depicted in Figure 5E, to characterize the impedance amplitude, membrane capacitance and cytoplasm conductivity of non-small-cell lung cancer cell lines (95D, 95C, A549, and H1299) [48]. Membrane capacitance was used to distinguish between high metastatic (95D) and low metastatic (95C) lung cancer cells and correlates with cell migration. Two oncogenes, cyclin Y (membrane protein) and cyclophilin A (cytosolic protein), were knocked down in 95D and A549 cells, respectively, using RNA interference. Membrane capacitance corresponded with cyclin Y expression and cytoplasm conductivity corresponded with cyclophilin expression. These studies demonstrate that EIS and IFC are sensitive enough to discern differences in human cancerous and normal cell populations.

#### Metastasis

Cancer cell metastasis is the spreading of malignant tumor cells throughout the body causing high mortality rates and is strongly associated with chemoresistance. EIS can be used to distinguish different stages of metastatic cancer. For instance, Huerta-Nunez et al. [60] used impedance to discriminate between metastatic (SK-BR-3) and non-metastatic breast cancer cells (MCF-7 and MDA-MB-231). Magnetic nanoparticles were used to isolate the cancer cells prior to the impedance analysis; thus, nanoparticles were included in each measurement. The impedance spectrums for each SK-BR-3, MCF-7, and MDA-MB-231 cells differed in the α-dispersion region between 1 kHz and 10 kHz where MDA-MB-231 < MCF-7 < SK-BR-3. The differences in the impedance was attributed to the structural heterogeneity of these breast cells. The measurements were completed at low cell concentrations, ~100 cells/mL, demonstrating the sensitivity of impedance-based sensing to detect metastatic breast cancer. These results show that impedance sensing has the capability to distinguish metastatic and non-metastatic breast cancer cells. This level of sensitivity displays that impedance may potentially be adequate for assessing liquid biopsies for circulating tumor cells (CTCs), a rare subpopulation of highly resistant cancer cells found in the blood of cancer patients with the ability to metastasize [61], and extracellular vesicles within the blood which both contribute to cancer cell heterogeneity and influence chemoresistance.

### 4.2. Cell Monitoring

Cancer cellular events such as adhesion, spreading, proliferation, and viability have been successfully monitored using ECIS. Cell proliferation is the process of cells dividing to increase the number of cells present. As cells proliferate in ECIS systems they cover the sensing electrodes allowing continual measurement overtime. When cells no longer proliferate, their viability begins to decrease, therefore impacting the measured impedance. Yang et al. [58] examined the differences between oral cancer cells (CAL 27) and non-cancer oral epithelial cells (Het-1A) during cell adhesion, spreading, and proliferation. These measurements were completed inside an incubator at 37 °C and 5% CO_2_ using a commercially available real-time cell analysis system (ACEA Biosciences Inc.) with circular electrodes. For CAL 27 and Het-1A cells, the impedance was reported as a cell index, a ratio of electrode resistance with cells to the electrode resistance without cells. During cell adhesion and spreading, the first 2 h of impedance measurement, Het-1A cells had a higher cell index than CAL 27 cells. Once the cells transitioned to the proliferative stage the cell index was higher for CAL 27 cells rather than the Het-1A cells. Cell viability before and after the impedance analysis remained intact (~90%). This study indicates that ECIS is suitable to detect cell spreading and proliferation and these cellular events are viable biomarkers to discern cancer and non-cancer cell populations. 

Single cell monitoring measurements have been completed on the epithelial mesenchymal transition (EMT) of A549 lung cancer cells [56]. EMT is linked to cancer metastasis and is the process epithelial cells undergo to exhibit mesenchymal cell phenotype in which they lose polarity and cell-cell adhesion, gain migratory, invasiveness, and resistance to apoptosis properties [23]. One important hallmark of EMT is resistance of tumor cells to anoikis [62], that is a unique mode of apoptotic cell death induced upon detachment due to insufficient cell-matrix interactions [63]. Induction of EMT has been associated in several cancers [64,65] with increased resistance to drugs such as cisplatin [66], gemcitabine [67], and therefore represents an attractive target in oncology. To monitor EMT, Zhao et al., improved on their IFC single cell microdevice system with a constriction to increase the throughput of single cell measurements from 450–650 cells total to 100 cells/s [56]. EMT was indicated in A549 cells via decreased membrane capacitance, decreased cytoplasm conductivity, and visible changes in cell morphology. This study indicates that IFC is suitable to detect cell phenotype change as they correspond to cancer progression.

Single cell monitoring measurements can be extremely useful to gain insight into the phenotypic changes associated with anoikis in cells freshly detached from the extracellular matrix. Recently, a microfluidic device was developed to separate dead cells and debris from viable cells in order to investigate the properties of these floating cells in terms of proliferation, structure, protein expression and chemoresistance [68]. While floating cells tend to form complex 3D floating structures (spheroids) it was recently reported that the formation of these 3D structures are not necessary for chemoresistance and that cell detachments, per se makes cells highly resistant to conventional anticancer drugs [9]. Indeed, microfluidic-isolated floating cells were 30-fold more resistant compared to adherent cells [68]. Coupling these findings with EIS or IFC microdevices will improve single cell monitoring of cancer cell plasticity as it relates to chemoresistance.

#### 4.2.1. Microenvironment 

In cancer research, the extracellular matrix is a major contributor in tumor metastasis [19,21,69]. Most culturing conditions are 2D and impedance is commonly measured on cells grown as an adherent layer. However, more researchers are moving toward using both 2D and 3D culturing conditions to mimic the cancer microenvironment in the body and better understand cancer cell dynamics [8,19,20]. The differences between cells grown in 2D and 3D culture have been explored, but the electrophysiological changes have not been fully characterized. 3D microenvironments are more representative of in vivo conditions allowing better study of anti-cancer drugs [9,70]. There are many 3D systems that can be studied: scaffold-based, including natural and synthetic polymers, and scaffold-free, consisting of spheroids and tumorspheres. Other factors, such as oxygen, pH, and nutrients, can change within the microenvironment [69,71]. These systems can be tested with impedance-based and dielectrophoresis-based microfluidic devices allowing for the examination of chemoresistance at different stages of cancer and the impact of the microenvironment [72]. 

Tran et al. [73] altered the microenvironment to mimic tissue structure within an ECIS microdevice by incorporating a hydrogel and observed the viability of cervical cancer (HeLa) cells when treated with doxorubicin and 5-fluoracil. The hydrogel was made of 1% agarose gel prepared in phosphate buffer solution and the microdevice contained eight castellated microelectrodes in the impedance sensing region. To test the impact of the hydrogel, HeLa cells were seeded inside the microdevice, allowed to form a monolayer, after which the hydrogel was introduced to fill the space between the monolayer and the top surface of the microdevice. Solutions containing doxorubicin and 5-fluoracil were circulated through the device and impedance was monitored via normalized resistance. It was found that the hydrogel created concentration gradients for both drugs, and doxorubicin was more toxic to the HeLa cells, as indicated by a significant decrease in normalized resistance after 24 h (100% to ~15%). Moreover, 5-fluoracil also decreased the normalized resistance of the HeLa cells over a longer time frame, i.e., after 72 h normalized resistance reduced from 100% to ~25%. Cell viability was estimated using the ratio of relative impedance drug treated to untreated HeLa cells. This study is novel due to the coupling of a hydrogel in ECIS measurements and notably the sensitivity of the electrodes was not negatively impacted by the hydrogel.

Additionally, Mansoorifar et al. [74] used EIS and dielectrophoresis to assess the impedance, membrane capacitance, and cytoplasmic resistance of human prostate cancer cells (PC-3) exposed to microenvironment changes in pH. Cells were tested in a microwell device containing square electrodes on the top and bottom of the microwells and in low conductivity buffer solution. The dielectrophoresis force was used to trap cells in the microwells between the electrodes and the impedance was measured over time as the pH was changed from 7.3 to 5.8. It was found that rapidly changing pH decreased the membrane capacitance and increased the cytoplasmic resistance within 4 min of the pH change. A change in external pH was assessed due to the changing extracellular pH surrounding cancerous cells, which impacts their growth rate and metabolism [74,75]. It is known that acidic microenvironments are important not only in the progression and spread of aggressive cancer phenotypes, but also play a role in supporting the efficacy of drugs to target cancerous cells [76,77]. Therefore, changes in pH may impact cancer cell plasticity contributing to changes in chemoresistivity. 

#### 4.2.2. Impedance Coupled with Dielectrophoresis (DEP)

Coupling EIS with dielectrophoresis (DEP) creates a more powerful bioanalytical tool which provides extra information about cancer cell dielectric properties and correlations to their biological meaning can be made. While EIS and DEP both measure dielectric behavior of cells more information is gained because the movement of cells can be monitored via recorded videos and cells can be patterned in an organized manner. DEP methods have been coupled with impedance for cancer cell monitoring and characterization. EIS and DEP are label-free electric field-based technique that can exploit the differences amongst the dielectric properties of cancer cells [78]. DEP is similar to EIS because cell polarization is utilized to examine the dielectric properties of cells and has been used for bioparticle characterization [79], manipulation [80], and sorting [81]. DEP has been implemented to isolate rare CTCs [53]. However, we highlight a paper that uses both EIS and DEP for the manipulation and characterization of lung cancer cells [82]. 

**Figure 6:**
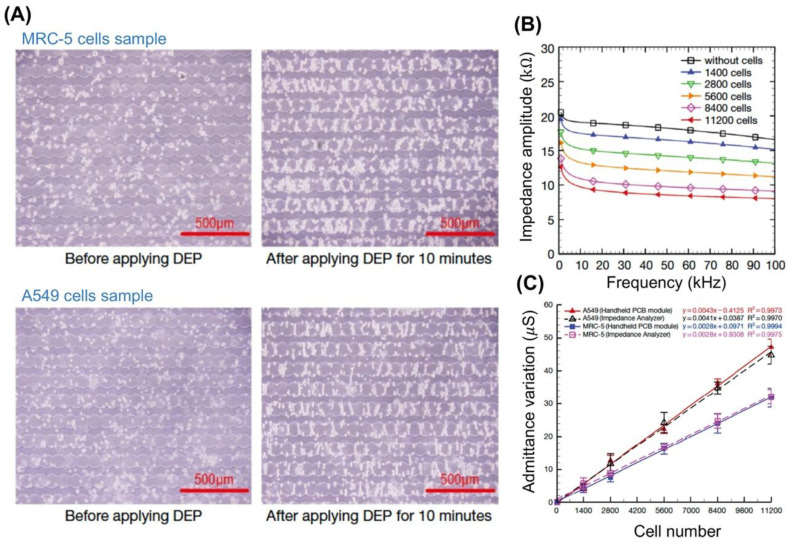
(**A**) DEP patterning of human lung carcinoma (A549) and lung epithelial cells (MCR-5). (**B**) Impedance spectrums for A549 cells at various cell concentrations. (**C**) Experimental data with trendlines for admittance measurements for A549 and MCR-5 cells. Reprinted with permission from [82].

Nguyen et al. [82] used EIS and DEP simultaneously to characterize human lung carcinoma (A549) and human lung epithelial cells (MCR-5). A handheld electronic module including a power supply, wave generator, and pre-amplifier was developed and combined with the microdevice to complete the impedance measurements. Circular interdigitated electrodes were used to trap A549 and MCR-5 cells, Figure 6A. For the impedance measurement, cell concentration was varied from 1400 to 11,200 cells, and the magnitude of the impedance decreased with increased frequency, Figure 6B. A549 and MCR-5 cells were distinguished based on the variation in their admittance (synonymous to conductance), Figure 6C. This unique system, handheld electronic module plus microdevice, is beneficial because of its easy use, good sensitivity, low cost, and portability. Advantages to combining EIS and DEP include tight control of cell movement, cell patterning, reduced analysis time due to cells being quickly trapped via the DEP force, and enhanced sensitivity. These electrokinetic techniques combined show promise in single cell analysis to identify and determine the time frame in which cancer cells plasticity changes (i.e., cancer cells become chemoresistant). 

Quantifying the dielectric properties of cancer cells is an important step in monitoring and understanding cancer cell dynamics (plasticity and chemoresistance). In order to identify plasticity changes, which correlates to cells chemoresistance, baseline dielectric properties should be established such that alternative methods for chemotherapy treatments can be developed and to advance the field of cancer biology. EIS is appropriate to detect cancer cell spreading and proliferation and will be beneficial in detecting changes in plasticity. When cancer cells become chemoresistance their change in state is associated with slow proliferation and quiescence [83]. The slowness in proliferation may be unique to different cancer cell populations and detectable with impedance sensing. Coupling drug monitoring with cancer cells microenvironment, can support further exploration for understanding cancer cell dynamics and chemoresistance.

### 4.3. Drug Monitoring

Examining the efficacy of drug concentrations and toxicity on cancer cells is essential for identifying changes in cancer cell plasticity and chemoresistance. Drug monitoring can help clinicians better understand chemoresistance and its time scale, one of the major obstacles in treating cancer that can cause recurrence, even after remission. Impedance does not compromise cell viability and provides a real-time measurement of cancer cell dynamics [84,85]. This is important in order to monitor the cells response to chemotherapy drug treatments. Viability is an important aspect for studying the effects of drug cytotoxicity and the response of cells to changes in their microenvironment. Apoptosis and necrosis contribute to reduced cell viability. Apoptosis, also known as “cell suicide”, is the process in which cells are programmed to be removed in order to maintain the overall health of the organism. Necrosis differs because cells die due to injury or other external factors [14]. Apoptosis and necrosis are important in understanding chemoresistance and the removal of dead cells from these processes will impact impedance. IFC, ECIS, and EIS have all been shown to be helpful with obtaining information about cancerous cells after drug treatment, either as a timed study or at the molecular level. 

Apoptosis and necrosis have been monitored using ECIS [14,86]. Arias et al. [43] measured the effects of drug uptake, toxicity, and inhibitors on oral squamous cell carcinoma cells (CAL 27). The ECIS device used is the commercially available real-time cell electronic sensing system from ACEA Biosciences, which utilized circular microelectrode arrays for impedance sensing. The anti-cancer drug cisplatin causes cell apoptosis and was added to the CAL 27 cells at concentrations ranging from 5 µM to 25 µM. It was observed that higher doses of cisplatin decreased the cell index after the initial increase from cell spreading. In addition, nicotine was combined with cisplatin to inhibit cell apoptosis resulting in a prolonged increase in the cell index due to proliferation and then a decrease in the cell index due to apoptosis [43]. This real time monitoring of CAL 27 cells spreading, proliferation, and apoptosis provides information about cell viability and chemoresistance. 

Also, Xu et al. [87] looked at the effects of cisplatin on four different cancer cell lines, CaSKi (cervical), HeLa (cervical), RKO (colon), and SMMC-7721 (liver), with a novel ECIS microfluidic device. This device contains four major components: a printed circuit board, a glass substrate with interdigitated electrodes for ECIS, a layer of polydimethylsiloxane (PDMS) with eight chambers to seed cells on top of the electrodes, and an additional layer of PDMS with christmas tree structure microchannels to produce concentration gradients. Cancer cells were seeded in the chambers using fluid flow and allowed to become adherent on the electrodes and then concentration gradients of cisplatin were formed. Air bubble valves were used to stop fluid flow from the christmas tree microchannel to create stagnant concentration gradients. Similar to Arias et al. [33] it was shown that higher doses of cisplatin decreased cell impedance. ECIS measurements revealed that CaSki and SMMC-7721 cells were most sensitive to the cytotoxic effect of cisplatin with significant decreases in the normalized impedance after 24 h compared to decreases in normalized impedance after 48 h for the HeLa and RKO cells. This novel device facilitated a quick assessment of cisplatin’s cytotoxic effect on a variety of cancer cells, which is desirable in drug discovery efforts that targets CSCs. 

### 4.4. Single Cell Analysis

Single cell impedance sensors allow for studying the heterogeneity of cell populations, which is important for understanding cancer cell dynamics. Typically, PCR or flow cytometry is used to study single cells, however, these methods may have low throughput and require biomarkers. Han et al., used an EIS system to characterize whole cells at the single cell level [5]. EIS was used to differentiate between different stages of cancer, which allowed for the examination of the heterogeneity of tumor cell populations for diagnostic purposes such as detecting CTCs. Normal (MCF-10A), early state MCF-7), invasive (MDA-MB-231), and metastasized (MDA-MB-35) breast cancer cell lines were used to determine if impedance is sensitive enough to discriminate stages of cancer in which membrane capacitance and resistance were calculated from the impedance data. An average of 7–10 cells were tested, and it was found that the magnitude and phase were different for each cancer pathologic state. The membrane capacitance was lower for the metastasized breast cancer cells corresponding to a compromised more permeable cell membrane. While the change in membrane resistance was not as distinguishable amongst the breast cancer cells. This research group demonstrated that impedance spectroscopy can discriminate the various stages of breast cancer, therefore providing a tool for investigating the heterogeneity and progression of cancer at the single cell level [5].

Cho et al. [51] also used EIS to study metastatic head and neck cancer cells at the single cell level. The microdevice included 16 microarrays for analysis, where the impedance was monitored at each microarray site. Two 16 microarray devices were tested, one with 2 µm electrode spacing and the other with 4 µm spacing. The impedance spectra of a poorly metastatic cell line (686LN) and highly metastatic cell line (686LN-M4e) was generated. To trap each single cell, a negative pressure was applied and manually controlled, and the impedance was obtained over a frequency range of 40 Hz to 10 MHz; approximately 50 to 80 single cells were tested per sample. The resulting spectra displayed a higher impedance phase value for 686LN and a lower impedance phase value for 686LN-M4e, confirming that impedance spectroscopy can be used to identify and discriminate single cells of head and neck cancer. The microdevice with the smaller electrode spacing increased the sensitivity of the impedance measurements for the 686LN-M4e cells. From the statistical analysis, it was found that the impedance phase values of the two cell lines were statistically different, which can be attributed to differences in cell morphology, protein and gene expression. However, the magnitude of the measured impedance values were not statistically significant, and as such did not distinguish the two cell types. These impedance results were reproducible such that 686LN and 686LN-M4e cells tested at different time points generating two batches yielded the same trend (686LN has higher characteristic impedance phase value statistically different compared to 686LN-M4e). Single cell analysis is important in the detection and quantification of heterogeneity within cancer cell populations. 

The scientific reports highlighted here are important in the examination of cancer cells, their plasticity, heterogeneity, and pathological state as it corresponds to chemoresistance. To recap, impedimetric biosensors are capable of distinguishing different types and stages of cancer for single cells or many cells. Impedimetric biosensors are good tools for monitoring dynamic cellular processes like cell adhesion, spreading, proliferation, EMT, and drug induced apoptosis. Lastly, coupling impedimetric sensing with DEP allows for controlled cell movement and patterning. Table 1 summarizes cell characterization, cell monitoring, drug monitoring, and single cell impedance studies using ECIS, IFC, and EIS microdevice systems. 

These basic laboratory findings (i.e., impedance used as a tool to characterize and monitor cancer cells) encourage the application of microfluidic-based single cell analysis and sorting in translational oncology [99] for instance for the isolation and characterization of CTCs. It is important to mention that due to the plastic properties of cancer cells CTCs should be analyzed immediately after isolation since expansion of these cells may provide misleading results. For instance, it is known that cancer cells growing under adherent conditions, that are highly sensitive to anti-cancer drugs, become highly resistant when growing under floating conditions and rapidly become sensitive when cells are grown back under adherent conditions [8].The high degree of plasticity in terms of chemoresistance displayed by cancer cells limits the time window and amount of biological material available for analysis and it is particularly important in the CTCs field where only few cells, in the range of 1–10 CTC/mL [100] can be obtained. We have highlighted three impedimetric studies that have positive implications for CTCs. Thus, in a clinical setting microfluidic-based technology for CTCs will have practical and widespread applications in personalized medicine for early tumor diagnosis, prognosis, as well as for monitoring responses to chemotherapy drugs.

## 5. Future Trends in Monitoring Cancer Cell Dynamics

EIS, ECIS, and IFC have been highlighted as methods for cell characterization and monitoring due to their sensitivity and specificity for discrimination between normal cells, cancerous cells, and different stages of cancer. While impedance sensing is a valuable tool for monitoring cancer cell adhesion, spreading, proliferation as well as distinguishing multiple types of cancer cells, other aspects of cancer cell dynamics such as plasticity and heterogeneity need to be considered during monitoring. Cancer cell phenotypes can be correlated with intrinsic dielectric properties such as impedance, capacitance, permittivity, and conductivity and electrode-based platforms can quickly identify cancer cell populations in research and clinical settings. Thus, impedance sensing can be used as a tool for monitoring cancer cell heterogeneity and changes in plasticity to identify cells that will switch from non-resistant to chemoresistant (i.e., CSCs) prior to drug treatment [9]. This approach would greatly improve chemotherapy treatments by allowing researchers and clinicians to optimize and monitor drug treatment strategies that will target cells that switch phenotypes [83]. As mentioned above, examination of the microenvironment will be crucial in studying cancer cell dynamics to better mimic in vivo conditions [69,70,71]. The cell patterning capabilities of EIS combined with DEP can be employed to build complex 3D microenvironments that represent in vivo cancer conditions. Another aspect of DEP is its implementation to continuously sort a variety of cells [81,101,102,103,104], thus an exciting possibility is to sort CSCs from non-CSCs prior to impedance monitoring. These techniques can also be used for drug optimization and monitoring as well as to understand the molecular and environmental driving forces involved in plasticity. These strategies can be extended to personalized chemotherapy medicine in which patient-specific cancer cells can be isolated, analyzed via EIS and DEP, or built into screening platforms to assist clinicians in determining the best treatment plan.

Furthermore, mathematical modeling of equivalent electrical circuits and plasticity facilitated with microfluidic platforms can expand our knowledge of cancer cell dynamics. Thus far, we have focused on bioanalytical tools that are able to measure the dielectric properties of cancerous cells in order to understand their dynamics in terms of adhesion, spreading, proliferation, and heterogeneity and ultimately plasticity and chemoresistivity [105]. Mathematical modeling of plasticity helps to predict the response of cancer cells to chemotherapy drugs. EIS measurements can assist in modeling chemotherapy drug dosage regiments for cancer patients using pharmacokinetic pharmacodynamics mathematical modeling. The simplest model would be to treat each microenvironment as a stirred tank reactor and modify the modeling equations to include growth and maintenance kinetics. The information fed into the model would originate from data collected by experiments performed at a 2D level and then later in a 3D extracellular matrix [106,107]. Impedance measurements can provide continuous growth measurements without disturbing the cells allowing all kinetic and maintenance parameters for the various phenotype models to be fitted. It is critical to include all parameters that modify both the intrinsic and extrinsic properties for the cancer cells that will affect cancer cell plasticity (obtained from impedance sensing measurements). Thus, early phenotype detection from EIS and DEP measurements is an important area for exploration due to the techniques being non-invasive. Mathematical modeling can serve as a future tool for early detection and prediction of when cells have begun to switch phenotypes [106,108]. Modeling has become increasingly important and can serve as another tool in combination with bioanalytical methods for studying cancer cell dynamics and chemoresistance. 

## 6. Conclusions

In this review, past and recent advances in impedimetric biosensors were discussed as tools for cancer cell monitoring. Currently, there are few methods for assessing the behavior and efficacy of chemotherapeutics via direct cancer cell analysis and modeling. On the cellular level, many of the techniques used to assess chemotherapeutics are histological, PCR, fluorescent labeling, and genomics. These processes are time consuming and destructive to cells. Using a non-invasive and quick method to study the electrical behavior of cancer cells and potentially monitor phenotype changes and chemotherapeutics can aid in decreasing mortality rates. The field of electrokinetics is emerging and has shown much promise for the characterization of impedance and membrane capacitance of cancerous cells. 

While the impedance measurement technique should be taken into consideration for different cell culture applications, it is possible to develop an EIS system that includes microenvironment, specialized electrode configuration, and a coupled method of trapping electrodes to fully understand the characteristic properties of cancerous cells. 2D and 3D culturing environments are highly important for understanding cancer cell dynamics because it mimics the microenvironment within the body compared to 2D cell culture systems that may not provide complete information about cell characteristics. The importance of testing 3D non-scaffold environments and natural extracellular matrices can possibly replace human testing, thereby advancing the process of studying chemotherapeutics and chemoresistant cancer. This should be considered for future EIS system measurements. From this review, it can be inferred that electrical impedance spectroscopy can be used as a tool to detect chemoresistant cancer, therefore allowing for finding better treatment options, advancements in oncology, and understanding cancer cell dynamics. This novel approach will broaden the capabilities for cancer detection as well as further solidify electrical impedance spectroscopy as an engineering tool for medical applications.

## Figures and Tables

**Table 1:** Summary of impedance spectroscopy for cancer cell analysis.

Type of Monitoring	Cancer Cell Line	Summary	Platform	Operating Conditions	Ref
**Cell Characterization**	PC-3, RWPE-1	Discerned and detected differences in impedance values for RWPE-1 and PC-3 at 8.7 kHz	EIS	Frequency: 100 Hz–1 MHz Voltage: NR Temperature: 25 °C	[57]
	HeLa, A459, MCF-7, MDA-MB-231	Distinguished stages of breast cancer and cell type based on response to electric field revealing different impedance outputs	EIS	Frequency: 20–101 kHz Voltage: 0.1–1.0 V	[88]
	MDA-MB-231, MCF-7, MCF-10A	Measured cell membrane differences indicated by membrane capacitance and cytoplasmic conductivity	EIS	Frequency: 3 kHz–3 MHz Voltage: NR Temperature: 37 °C	[50]
	CE81T CE81T-4	Cell densities compared to find the lower limit of detection Cytological stages were distinguishable based on admittance values	EIS	Frequency: 4 kHz Voltage: 1 V	[89]
	A549, MRC-5	Cell number and cell type detected based on admittance (inversely proportional to impedance)	EIS	Frequency: 1–100 kHz Voltage: 100 mV	[82]
	SV-HUC-1, TCCSUP	Cells were found to have distinct impedance values at 119 kHz	EIS	Frequency: 5 Hz–1 MHz Voltage: 0.5 V	[59]
	95C, 95D, A549, H1299	Cells distinguished by metastasis, oncogene cylin A, and oncogene cyclophilin A based on membrane capacitance and cytoplasm conductivity	IFC	Frequency: 1 kHz and 100 kHz Voltage: NR	[48]
**Cell Monitoring**	PC-3	Impedance was used to observe variations in cells dielectric properties with a change in pH (microenvironment change)	EIS	Frequency: 10 kHz–40 MHz Voltage: 0.2 V	[74]
	CAL27, Het-1A	Impedance discerned the spreading, adhesion, and proliferation of cells cultured directly on electrodes	ECIS	Frequency: 10 kHz, 25 kHz, 50 kHz Voltage: 10 mV	[58]
	H1299 A549, HeLa	H1299 distinguishable from HeLa via lower membrane capacitance and higher cytoplasm conductivity Epithelial-mesenchymal transition (EMT) discernible in A549 cells via higher membrane capacitance and cytoplasm conductivity (single cell monitoring achieved)	IFC	Frequency: 100 kHz, 250 kHz Voltage: NR	[56]
	MRC-5, QUDB	Impedance used to discern cell types, attachment, spreading, and proliferation properties	ECIS	Frequency: 200 Hz–150 kHz Voltage: 400 mV	[90]
	T24, TSGH8301	Different stages of bladder cancer discernable using impedance and lower grade bladder cancer had higher impedance than higher grade bladder cancer	EIS	Frequency: 1 kHz–100 kHz Voltage: 1 V	[91]
	T47D	Studied spreading of adherent cells and effects of ZD6474 (anti-cancer) drug treatment Impedance decreased with increased drug dosage indicating increased cell death	ECIS	Frequency: 100 Hz–1 MHz, 10 kHz (fixed) Voltage: 10 mV	[92]
	KYSE 90	Tested effects of cisplatin (anti-cancer drug) on cells Cisplatin induced cell morphology changes and apoptosis indicated by decreasing normalized impedance	ECIS	Frequency: 1 Hz–1 MHz Voltage: 10 mV	[93]
	HeLa	Viability of cells monitored with drug treatments of doxorubicin and 5-fluoracil Microenvironment manipulated with hydrogel to create concentration gradients and mimic tissue structure Both drugs decreased cell viability indicated with reduced normalized resistance	ECIS	Frequency: 4 kHz Voltage: 10 mV_pp_	[73]
**Drug Monitoring**	CAL27	Cell index (measure of impedance) distinguished between cisplatin (apoptosis inducer), nicotine (apoptosis inhibitor), and cisplatin + nicotine treatments	ECIS	Frequency: NR Voltage: NR	[43]
	CaSKi, HeLa, RKO, SMMC-7721	Implemented novel device with fluid mixing microchannels, air-bubble valves, and interdigitated microelectrodes to monitor cisplatin cytotoxicity and dosage dependent response detected via impedance	ECIS	Frequency: 60 kHz (fixed) Voltage: NR	[87]
	MCF-7	Cells treated with anti-cancer drug Chitosan-P to study effects on impedance Largest change in impedance observed at 21.4 kHz and impedance decreased after Chitosan-P treatment due to cell death	ECIS	Frequency: 10 Hz–100 kHz Voltage: NR	[94]
	MCF-7, MCF-7 WT, MCF-7 DOX	Cells types had higher resistive values based on drug resistance, concluding that impedance can distinguish between drug resistant phenotypes	ECIS	Frequency: 100 Hz–2 MHz Voltage: NR	[12]
**Single Cell Analysis**	MCF-7	Cell death monitored by the impedance of cells trapped on rough surface and treated with paclitaxel and mebendazole anti-tublin drugs at low and high doses Roughened and smooth electrode surfaces were compared to improve sensitivity of impedance readings and demonstrate the importance of nanoscale geometry Nano-roughened electrodes had a 20% greater sensitivity	EIS	Frequency: 0–60 kHz, targeted frequency at 4 kHz Voltage: 40 mV	[95]
	HeLa	Electrical properties of cells observed for 24 h period Cell shape change at 15 h marked by impedance characteristics based on cell spreading and adhesion to electrodes After 15 h membrane capacitance and cytoplasm resistance decreased	ECIS	Frequency: 10–100 kHz Voltages: 0.7 V and 0.9 V	[96]
	HeLa, HepG2, A549	Membrane capacitance and cytoplasm conductivity used to characterize three different cancer cells lines	IFC	Frequency: 10^3^ Hz–10^6^ Hz Voltage: 1 V	[97]
	686LN, 686LN-M4e	Highly metastatic and poorly metastatic head and neck cancer cell lines were measured on a 16-array microsystem The impedance spectra displayed a higher value for 686LN and a lower value for 696LN-M4e confirming the presence of different cells	EIS	Frequency: 40 Hz–10 MHz Voltage: 500 mV	[51]
	U87MG	Cells were grown on electrodes and treated with chlorotoxin (ion inhibitor) to monitor real-time shape changes and impedance changes	EIS	Frequency: 500 Hz–20 kHz Voltage: 10 mV	[98]
	MCF-10A, MCF-7, MDA-MB-231, MDA-MB-435	Magnitude and phase of impedance, membrane capacitance, and resistance differentiated each cell line, which represents stages of cancer	EIS	Frequency: 100 Hz–3 MHz Voltage: NR	[5]

## Abbreviations

CCSCT	Classical Cancer Stem Cell Theory
CSC	Cancer Stem Cell
CTC	Circulating Tumor Cell
DEP	Dielectrophoresis
ECIS	Electrical Cell-Substrate Impedance Sensing
ECM	Extracellular Matrix
EGF	Epidermal Growth Factor
EIS	Electrical Impedance Spectroscopy
EMT	Epithelial Mesenchymal Transition
IFC	Impedance Flow Cytometry
MSCs	Mesenchymal Stem Cells
PCR	Polymerase Chain Reaction
PDGF	Platelet Derived Growth Factor
SPM	Stemness Phenotype Model
TGF-α	Transforming Growth Factor-alpha
